# Ibrutinib in the Treatment of Solid Tumors: Current State of Knowledge and Future Directions

**DOI:** 10.3390/cells11081338

**Published:** 2022-04-14

**Authors:** Katarzyna Szklener, Adam Michalski, Klaudia Żak, Michał Piwoński, Sławomir Mańdziuk

**Affiliations:** Department of Clinical Oncology and Chemotherapy, Medical University of Lublin, 20-090 Lublin, Poland; michalski.g.adam@gmail.com (A.M.); zakklaudia3@gmail.com (K.Ż.); michalpiwonski2@gmail.com (M.P.); slawomir.mandziuk@umlub.pl (S.M.)

**Keywords:** ibrutinib, BTK inhibitor, Bruton’s tyrosine kinase, cancer, solid tumor

## Abstract

Bruton’s Tyrosine Kinase (BTK) is considered crucial in the activation and survival of both physiological and malignant B-cells. In recent years, ibrutinib, an oral BTK inhibitor, became a breakthrough therapy for hematological malignancies, such as chronic lymphocytic. However, ibrutinib’s feasibility might not end there. Several other kinases with established involvement with solid malignancies (i.e., EGFR, HER2) have been found to be inhibited by this agent. Recent discoveries indicate that BTK is a potential anti-solid tumor therapy target. Consequently, ibrutinib, a BTK-inhibitor, has been studied as a therapeutic option in solid malignancies. While most preclinical studies indicate ibrutinib to be an effective therapeutic option in some specific indications, such as NSCLC and breast cancer, clinical trials contradict these observations. Nevertheless, while ibrutinib failed as a monotherapy, it might become an interesting part of a multidrug regime: not only has a synergism between ibrutinib and other compounds, such as trametinib or dactolisib, been observed in vitro, but this BTK inhibitor has also been established as a radio- and chemosensitizer. This review aims to describe the milestones in translating BTK inhibitors to solid tumors in order to understand the future potential of this agent better.

## 1. Introduction

In recent years, not only has cancer been recognized as one of the major causes of death worldwide, but its incidence and mortality rate have grown rapidly [1,2,3]. The reasons behind that are complex and multifactorial. Still, they reflect the growth and aging of the worldwide population, as well as the increase in prevalence and distribution of several cancer risk factors [3,4]. Unfortunately, predictions for the future remain grim—the authors of the GLOBOCAN 2020 study predict 28.4 million new cancer cases occurring in 2040, an astounding increase of 47% from the corresponding 19.3 million new cases in 2020. Therefore, it is imperative to find new treatment options for this growing population of patients [3]. Although, currently, a plethora of studies researching new treatment methods are being conducted, we should also consider other possibilities—repurposing already established medications.

**Figure 1:**
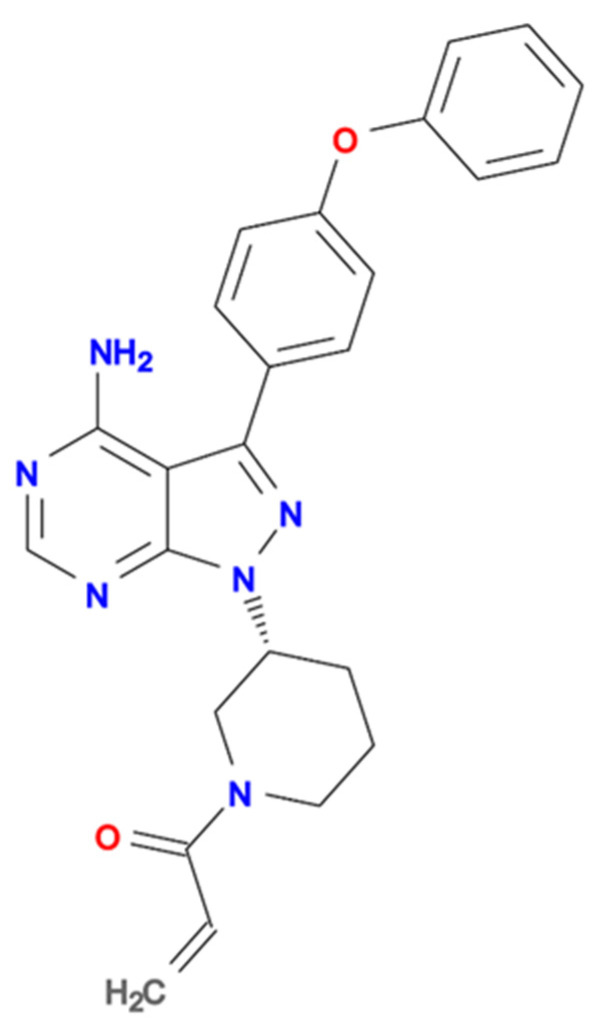
Ibrutinib; orally bioavailable, irreversible inhibitor of Bruton’s Tyrosine Kinase; molecular formula C25H24N6O2; chemical name 1-[(3R)-3-[4-amino-3-(4-phenoxyphenyl)pyrazolo[3,4-d]pyrimidin-1-yl]piperidin-1-yl]prop-2-en-1-one; molecular weight-440.50 Da [10,11].

Ibrutinib (Figure 1), also known as PCI-32765, is a first-of-its-kind agent, irreversibly inhibiting Bruton’s Tyrosine Kinase (BTK) [5]. As BTK is a critical player in B-cell receptor (BCR) signaling, ibrutinib was initially developed as a treatment option for the malignancies of the B-cell lineage [6]. However, BTK is not limited to B-cells—it is expressed in all hematopoietic lineages [7,8]. Furthermore, BTK has been found to play a crucial role in the tumor microenvironment—a complex and meticulous network of many types of cells and their precursors, such as pericytes, smooth muscle cells, fibroblasts of various phenotypes, myofibroblasts, neutrophils, eosinophils, basophils, mast cells, T-cells, B-cells, natural killer (NK) lymphocytes, as well as antigen-presenting cells such as macrophages and dendritic cells. All these cells take part in the pathophysiology of cancer [9]. These observations consequently make BTK a potential target in the treatment of solid tumors. Furthermore, ibrutinib is not entirely selective towards BTK—it has been discovered that over ten other kinases are inhibited by this drug, including those commonly associated with several solid tumors [5]. Taking advantage of the aforementioned aspects of both BTK biology and the non-selectiveness of ibrutinib, several studies have been conducted focusing on indication characteristics other than hematological malignancies.

This paper reviews the rationale behind using ibrutinib as a therapeutic option in solid tumors, as well as the currently available preclinical and clinical studies focused on utilizing ibrutinib in solid tumors.

## 2. Rationale behind Using Ibrutinib in Solid Tumors

### 2.1. Ibrutinib Targets BTK in Anti-Solid Tumor Therapy

BTK is a member of a TEC family of kinases (TFKs), a group of non-receptor kinases composed of BTK and four other kinases: tyrosine kinase expressed in hepatocellular carcinoma (TEC), IL-2 inducible T-cell kinase (ITK), resting lymphocyte kinase (RLK), and the bone-marrow tyrosine kinase gene on chromosome X (BMX) [12]. With the exception of RLK, a non-classical TFK, all of them are characterized by the presence of a pleckstrin-homology domain (PH), a domain exclusive of TFKs, responsible for binding the phosphatidylinositol-3,4,5-trisphosphate (PIP3) [12,13]. Following PH, there is the TEC-homology domain, containing one or two proline-rich regions, the SRC-homology 3 (SH3) and 2 (SH2) domains, and the tyrosine kinase region located at the carboxy-terminal end of those kinases [13,14].

The BCR signaling pathway is the pathway that BTK is probably most commonly associated with. Through the series of events, BCR activation results in the activation of the B-cell, thus enabling its differentiation and proliferation [15]. Nevertheless, BCR IgM itself is unable to exert its action—its proper functioning requires a non-covalent binding with disulphide-linked Igα (also known as CD79A)–Igβ (also known as CD79B) heterodimer [16]. This heterodimer carries an immune-receptor tyrosine-based activation motif (ITAM), containing two tyrosine residues [15]. As the antigen binds to the BCR, BTK activation begins—first, BTK gets phosphorylated at Y551 within its catalytic domain by either SYK, LYN or SRC kinases. Next, Y551 promotes the catalytic activity of BTK, resulting in its autophosphorylation at Y223 in its SH3 domain [15,17]. BTK is responsible for the subsequent phosphorylation of the phospholipase C-γ (PLCγ2), that stimulates a positive feedback loop [15]. Diacylglycerol (DAG) and inositol triphosphate (IP3), cleaved by the PLCγ2, activate the transcription of the nuclear factor of activated T-cells (NFAT) [15,18]. Lastly, autoantigens have been observed to drive the BCR-dependent activation of nuclear factor-κB (NF-κB) through a series of events involving SYK, PKCβ and BTK [15,19]. Notably, both NFAT and NF-κB have been proven to be involved in the promotion and progression of several solid tumors [20,21]. Furthermore, BTK is involved with protein kinases such as the extracellular signal-related kinases (ERK) and c-Jun N-terminal kinases (JNK), both of which have been strongly associated with solid tumors as well [22,23,24,25,26]. 

While BTK is a critical player in BCR signaling, its role is not limited to just that pathway. It is well established that BTK participates in other types of signaling pathways, such as chemokine receptor signaling pathways [15]. In B-cells, BCR stimulation promotes chemokine receptor type 4 (CXCR4) internalization; furthermore, BTK might also be associated directly with CXCR4 and CXCR5 through the interaction with the heterotrimeric G protein subunits Gα and Gβγ [27,28,29,30,31]. Recently it has been discovered that C-X3-C Motif Chemokine Receptor 1 (CX3CR1) knockout mice are characterized by impaired BCR signaling, and BTK expression was proven to be caused by defects in actin remodeling—a process normally controlled by CX3CR1 [15,32]. Furthermore, mouse B-cells deficient in the function of BTK have also been found to lack C-X-C Motif Chemokine Ligand 12-chemokine receptor type 4 (CXCL12-CXCR4) and C-X-C Motif Chemokine Ligand 13-chemokine receptor type 5 (CXCL13-CXCR5) [33,34]. CXCR4 and CXCR5 have been recognized as essential players in cancer biology, with the CXCL12/CXCR4 axis playing a pivotal role in inducing metastases [35]. Nevertheless, the role of CXCR5 is considerably more complex. On the one hand, the CXCL13/CXCR5 axis might help tumor cells evade host immune surveillance via down- or negative-regulation of T effector cell-mediated antitumor immunity. On the other hand, CXCR5 was proven to be involved in the determination of the antitumor activity of CXCR5+CD8+ cytotoxic lymphocytes, which were shown to exhibit a more potent proliferative capacity, granzyme B production, as well as increased TNF-α and IFN-γ expression, thus causing cancer cells’ lysis of several solid tumor types, including colorectal cancer, pancreatic cancer or thyroid cancer, more specifically, as compared to the CXCR5-CD8+ lymphocytes [36,37,38,39,40,41].

**Figure 2:**
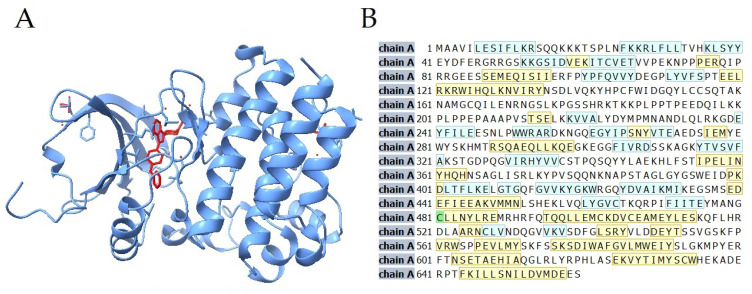
(**A**) Molecular structure of ibrutinib binding Bruton’s Tyrosine Kinase. Shown in color red is ibrutinib; blue represents the kinase [51,52,53]; (**B**) Sequence of Bruton’s Tyrosine Kinase. BTK is a 659 amino-acid protein. Ibrutinib inhibits BTK by irreversibly binding to cysteine-481, which is marked with the color green. Structure strands are marked with the color blue; structure helices are marked with the color yellow [54,55,56].

The discovery of a novel oncogenic isoform of BTK abundantly expressed in breast, ovarian, prostate and colorectal cancer has been another essential factor indicating the potential efficacy of BTK in the management of solid tumors. Its significance cannot be overlooked, as it is responsible for the therapeutic escape and protection of the cancer cells from apoptosis [42,43,44,45,46,47,48,49]. Furthermore, the overexpression of this BTK isoform on cancer cells has been associated with an increased expression of the genes with functions related to cell adhesion, cytoskeletal structure and extracellular matrix, as well as higher aggressiveness of cancer and a worse clinical outcome [43,50]. As discovered, the inhibition of this BTK isoform inhibited cancer cell growth and apoptosis and enhanced chemosensitivity, thus making targeting it an attractive opportunity for anti-cancer therapies [42,43,44,45,46,47,48,50] (Figure 2).

### 2.2. Ibrutinib’s Mechanism of Action as an Anti-Solid Tumor Drug

Several aspects of the biology of BTK, described previously, make it a potential target of anti-solid tumor therapy. Furthermore, even though ibrutinib is potent towards BTK, it is not entirely selective towards it [10,11,48] (Table 1). As ibrutinib exerts its action through binding with the cysteine residue 481 in the kinase domain of BTK, other kinases sharing similar cysteine residue might be prone to irreversible inhibition by ibrutinib [11]. Among such kinases, epidermal growth factor receptor (EGFR), human epidermal growth factor receptor 2 (ERBB2/HER2) and Janus kinase 3 (JAK3) can be found, which were proven to be associated with the development of solid tumors [57,58,59,60]. 

The mechanisms of action exhibited by ibrutinib include its ability to reverse the polarization of Th2 cells through inhibiting ITK—one of the kinases that show significant homology with BTK can be observed [61]. This reverse in polarization is possible due to a non-classical member of TFKs, RLK, on the Th1 cells, which allows for a proper maturation of the CD8+ lymphocytes, as RLK is not prone to inhibition by the ibrutinib [62,63,64]. This situation is not entirely welcome, as it might promote the pro-tumorigenic activity of the B-cells, as observed in melanoma, squamous-derived carcinomas, prostate and pancreas adenocarcinomas. However, BTK inhibitors disrupt B-cell activation and, thus, reverse some of those pro-tumorigenic effects [48,65,66,67,68].

**Figure 3:**
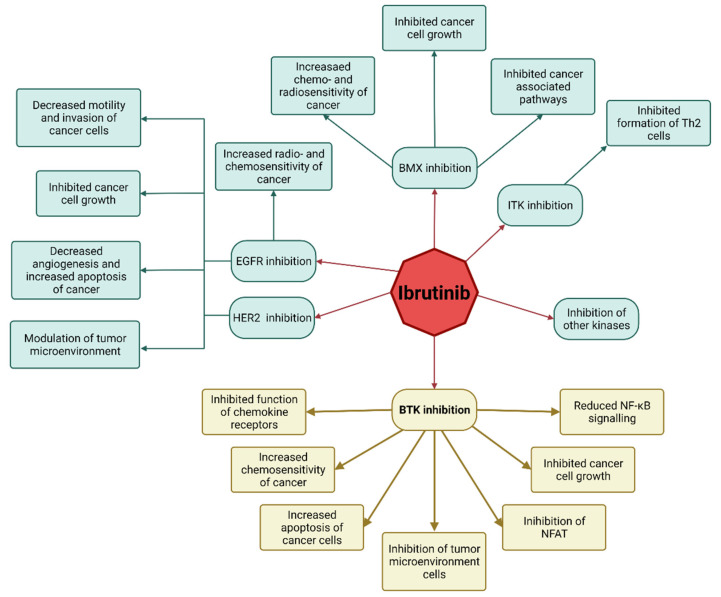
Ibrutinib as an anti-solid tumor drug. Ibrutinib exerts its action as an anti-solid tumor drug through its ability to inhibit both BTK and other cancer-associated kinases. In this figure, we focus on the kinases most relevant to the subject of this study: BMX, ITK, EGFR and HER2. BTK inhibition results in reduced NF-κB signaling, decreasing the promotion and progression of cancer cells and a reduced function of CXCR4 and CXCR5, leading to reduced cancer’s ability to induce metastases and evade host immunity, decreased cancer cell growth, increased apoptosis and chemosensitivity of cancer cells through the inhibition of novel, oncogenic isoform of BTK present on the cancer cells. The inhibition of BTK expressing tumor microenvironment cells (MDSC, mast cells and monocytes) results in the decreased survival of cancer cells. Through the inhibition of ITK, it causes a limited activation of Th2 cells, subsequently increasing the number of anti-cancer Th1 cells due to their expression of RLK, which is not expressed on Th2 cells [62,63]. The inhibition of BMX has been discovered to sensitize cancer to doxorubicin as well as chemo- and radiotherapy in prostate cancer; BMX-inhibition has been discovered to inhibit cancer cell growth in glioblastoma and to downregulate the activation of PI3K/Akt, STAT and NF-κB pathways [78]. Ibrutinib joins Cys797 residue of EGFR, thus inhibiting it, while HER2 inhibition occurs due to the key role of BTK in the AKT-ERK intracellular signaling. As a result, inhibition of EGFR and HER2 causes decreased angiogenesis, motility, invasion, and growth of cancer, while EGFR inhibition additionally increases the s radio- and chemosensitivity of cancer [48]. BTK, Bruton’s tyrosine kinase; EGFR, epidermal growth factor receptor; HER2, human epidermal growth factor receptor 2; ITK, Interleukin-2-inducible T-cell Kinase; JAK3, Janus kinase 3; NF-κB, nuclear factor kappa-light-chain-enhancer of activated B-cells; NFAT, nuclear factor of activated T-cells.

As mentioned previously, cells found in the tumor microenvironment play a crucial role in the initiation, development and dissemination of cancer. Although this meticulous network comprises numerous pathways, the BTK signaling pathway is among the most vital ones for cancer progression [69]. Myeloid-derived suppressor cells (MDSC) possess immunosuppressive properties and have been proved to be critical for tumor evasion mechanisms [70,71]. Importantly, MDSCs express BTK, which makes them prone to inhibition by ibrutinib, which decreases the immunosuppressive functions of MDSC and increases the levels of CD8+ lymphocytes [72,73]. Among other cells of the tumor microenvironment expressing BTK, we can find monocytes and mast cells [55,74]. This point is further confirmed as ibrutinib has been observed to lead to a significant decrease in TNFα, IL1β and monocyte chemo-attractant protein-1 (MCP1), as well as a reduction of the ability of mast cells to degranulate [75,76]. As a result, ibrutinib can cause decreased peritumoral fibrosis and tumor vascularization, leading to reduced tumor cell survival [48,76,77] (Figure 3).

## 3. Ibrutinib in Studies

This section describes current published studies regarding ibrutinib in both preclinical studies and clinical trials. The stock of the results for each tumor discussed is represented in Table 2.

### 3.1. Lung Cancer

Gao et al. [79] conducted an animal study including nude mice with xenograft tumors. Scientists used an H1975 cell line of non-small cell lung cancer (NSCLC), possessing an L858R/T790M mutation rendering the cancer erlotinib-resistant. Scientists compared survival times (ST) between three groups of mice—placebo, erlotinib- and ibrutinib-treated ones (both ibrutinib and erlotinib were obtained from Selleck Chemicals, Houston, TX, USA) observing a significantly increased ST in the group treated with ibrutinib—the mean ST in placebo and erlotinib-treated groups were both 17.8 days (95% confidence interval [CI] = 14.3 to 21.3 days), while the mean survival time in the ibrutinib-treated group was 29.8 days (95% CI = 26.0 to 33.6 days) [79].

Wu et al. [80] confirmed ibrutinib’s (Haoyuan Chemexpress Inc., Shanghai, China) activity towards the aforementioned cell line. However, the authors discovered that ibrutinib was highly active only against PC-9 (EGFR Del19) and H3255 (EGFR-L858R) cell lines (American Type Culture Collection, Manassas, VA, USA). In contrast, the activity against cells harboring a T790M mutation was only moderate. Furthermore, ibrutinib was not active against wild-type *EGFR* NSCLC cells, although the reason behind that difference has not been discovered. Lastly, trametinib, a mitogen-activated protein kinase (MEK) inhibitor, was discovered to potentiate ibrutinib’s effect against the T790M cells in vitro but not in vivo [80]. 

Fu et al. [81] decided to focus on different aspects of this subject, evaluating the role of ibrutinib in tumor cell-platelet crosstalk in lung cancer. As discovered, co-cultures of platelets and A549 cells (American Type Culture Collection, Manassas, VA, USA) increased the ability of cancer cells to proliferate, migrate and invade, while the administration of ibrutinib abolished these effects in just 48–72 h [81].

We believe that all three mentioned studies prove ibrutinib to be a potentially feasible option in some types of lung cancer. Interestingly, a therapy composed of ibrutinib with trametinib could be an interesting option for introducing this agent as a therapeutic option in NSCLC. Nevertheless, to take that approach from the bench to the bedside, the discrepancies between the in vitro and in vivo observations must first be addressed. Wu et al. [80] suggest that, as ibrutinib has been developed as a therapeutic option against hematological malignancies, current pharmacokinetics of this agent might be responsible for this difference. Thus, changes in the formulation of ibrutinib might be able to resolve this issue. 

Interestingly, the exact mechanism responsible for the synergism between ibrutinib and trametinib in the treatment of NSCLC is not yet fully understood. MEK-inhibitors have been discovered to be ineffective as a monotherapy for patients diagnosed with NSCLC [100,101,102,103], although capable of potentiating the effects of the therapy with EGFR-inhibitors on EGFR-inhibitor-resistant cell lines [104,105,106]. As one of ibrutinib’s potential mechanisms of action is the inhibition of the EGFR, this is most likely the reason behind the synergistic effect of these two compounds against the T790M cell line. Another mechanism of synergism might be related to the UDP-glucuronosyltransferase enzymes (UGTs). An overexpression of UGTs has been discovered in NSCLC, which is suspected to stimulate cancer progression [107]. As discovered by Korprasertthaworn et al. [108], both ibrutinib and trametinib are potent inhibitors of UGT1A1 and are also likely to inhibit UGTs 1A7-1A10, potentially serving as another mechanism of synergism between these two agents [108].

Zhang et al. [109] decided to focus not on ibrutinib itself but on its derivative—Ibr-7. In this study, the authors evaluated the activity of ibrutinib and Ibr-7 on four different cell lines—PC-9 line, H1975 cell line and two wild-type cell lines—A549 and H460 (Cell Bank of Type Culture Collection of the Chinese Academy of Sciences, Shanghai, China). The authors found Ibr-7 to exhibit an effect that is stronger than ibrutinib’s antiproliferative effect. Still, both ibrutinib and Ibr-7 could inhibit EGFR with half-maximal inhibitory concentration (IC_50_) values that can be achieved in vivo. Significantly, while ibrutinib decreased the phosphorylation of AKT and ERK, Ibr-7 dramatically suppressed downstream signaling, including mTOR, p-S6K and p-S6. While both of these drugs influence the activity of mTORC2, only Ibr-7 was capable of dephosphorylating mTORC1. Since the authors discovered that the inhibition of the EGFR was not necessary for the anti-cancer activity of Ibr-7, they proposed the mTORC1 inhibition to be a possible antitumor action of this derivative. It was also detected that the peak serum concentration (C_max_) of Ibr-7 is less than half of the ibrutinib; hence, the bioavailability of Ibr-7 leaves room for improvement. Therefore, it was proposed to apply molecular modification or biomaterial encapsulation as a possible solution to this issue [109]. Nevertheless, it is essential to mention that Ibr-7 is not approved for use on humans, thus making these observations interesting but clinically irrelevant.

To the best of the authors’ knowledge, the only clinical study regarding the efficacy of ibrutinib (IMBRUVICA^®^) in lung cancer is one published by Hong et al. [82]. In 2019 they described the effects of their phase 1b/2 clinical trial focusing on the effectiveness of ibrutinib in combination with PD-L1 inhibitor durvalumab in patients with pretreated stage-III/IV breast cancer, pancreatic adenocarcinoma and NSCLC. Unfortunately, as observed by the authors, the results of such a therapy were poor. Here, 0% of the patients diagnosed with NSCLC responded to an ibrutinib-durvalumab treatment, with the median overall survival (mOS) being 7.9 months (95% CI: 5.4–17.6 months) and the median progression-free survival (mPFS) being 2.0 months (95% CI: 1.7–4.0 months). As all (n = 28) NSCLC patients suffered the adverse effects (AE) of such a therapy, with the vast majority of them (n = 21; 75%) experiencing an AE of grade 3 or above, this therapy was deemed not only ineffective but also conducive to a wide variety of side effects [82].

We can observe an interesting discrepancy between the preclinical and clinical studies focusing on the feasibility of ibrutinib in NSCLC. Although the reasons behind that are yet to be discovered, we suspect that differences between the in vitro and in vivo pharmacokinetics of ibrutinib, found by Wu et al., are most likely involved. Furthermore, the population on which Hong et al. focused their studies did not necessarily represent the entirety of the population of patients diagnosed with NSCLC. Here, patients enrolled in this study have already failed a median of three prior lines of therapy. It might be speculated that as this population was drug-resistant, the results of the study focusing on the more general population might vary. Nevertheless, we believe that ibrutinib combined with other agents should be the main focus of the forthcoming studies. Inhibitors of PD-L1 might be an interesting option to be evaluated; as discovered by Sagiv-Barfi et al. [89], ibrutinib showed synergism when combined with this type of agent, albeit in other types of cancer. Lastly, ibrutinib has been observed to exert a significantly more potent activity towards mutated *EGFR* cell lines than the wild-type *EGFR* cell lines. As mentioned previously, the reason behind that difference is not yet understood, and should be thoroughly investigated in future studies. 

### 3.2. Endometrial Cancer

To the best of the authors’ knowledge, the only study focused on the feasibility of ibrutinib in endometrial cancer was the one conducted by Tamura et al. [83] in 2018. In their research, Bruton’s Tyrosine Kinase inhibitors (BTKi) were hypothesized to have an antiproliferative action. The authors evaluated an astounding number of 61 anticancer agents in cell growth inhibition studies, including ibrutinib (MedChemExpress, South Brunswick Township, NJ, USA). In the patient-derived tumor organoid (PDO) called REME9, established from a carboplatin/paclitaxel-resistant endometrioid adenocarcinoma with squamous differentiation, ibrutinib induced growth inhibition. By contrast, ibrutinib’s effect on REME16, an organoid established from an endometrioid, clear cell adenocarcinoma, was significantly less pronounced [83]. 

Therefore, we believe that the results of this study indicate the potential feasibility of ibrutinib as a treatment option in endometrial cancer, especially in adenocarcinoma with squamous differentiation. Nevertheless, it is too early to advance any firm statements, as more studies, especially clinical trials, are required. We propose that future researchers focus on different approaches, especially on the feasibility of ibrutinib as an element of polytherapy.

### 3.3. Ovarian Cancer

In 2015, Zucha et al. [84] investigated the role of BTK and BTKi in ovarian cancer, discovering a high level of expression of BTK in cancer cells in metastatic and late-stage disease. During the next stage of this study, the authors found that a higher expression of cancer stem cell (CSC) markers was associated with cell resistance to platinum-based drugs. As BTK is critical in regulating ovarian CSC, the authors hypothesized that BTK is a driver of resistance to platinum-based drugs. Indeed, the authors discovered that in cell lines resistant to this type of chemotherapy, ibrutinib (Cellagen Technology, San Diego, CA, USA) combined with cisplatin (Pharmachemie BV, Haarlem, The Netherlands) increased the latter’s efficacy, thus indicating the possible use of ibrutinib as a platinum sensitizer in this indication. Furthermore, ibrutinib has been found to decrease BTK phosphorylation and Sox2/Bcl-xL expression in malignant cells, diminishing their self-renewal capacities and proportion of CSCs. As such, the authors confirmed that the resistance to the platinum-based drugs relied on the overexpression of BTK [84,110].

Lohse et al. [85], in 2019, sought to investigate the efficacy of 30 different agents, including ibrutinib, on six patient-derived cell lines—two endometrioid, two clear-cell and two papillary-serous ones. Through ex vivo sensitivity testing, scientists discovered that the effect of ibrutinib on those cells varied greatly—while a weak inhibitory effect has been found in one papillary-serous and one endometrioid cell line, no effect has been discovered in clear cell lines, consistent with observations made by Tamura et al. regarding the endometrial clear cell adenocarcinoma [85,110].

Thus, further research should shift from evaluating ibrutinib’s feasibility as a monotherapy to its role as a part of polytherapy, especially as a chemosensitizer. Currently available studies do not provide enough data to make any firm statements regarding the efficacy of ibrutinib in ovarian cancer. As such, additional studies are required.

### 3.4. Breast Cancer

Grabinski and Ewald [86] conducted the first study evaluating the efficacy of ibrutinib in breast cancer. Authors focused on ibrutinib (Selleck Chemicals, Houston, TX, USA) and ibrutinib combined with dactolisib (Selleck Chemicals, Houston, TX, USA), a phosphoinositide 3-kinase/mammalian target of rapamycin kinase (PI3K/mTOR) inhibitor, on ten different breast cancer cell lines in vitro. Authors discovered that the results of such therapy vary depending on the exact investigated cell line. A significant reduction in HER2+ cells was demonstrated, while ER+ and triple-negative cell lines did not exhibit considerable attenuation due to such therapy. Authors discovered a decreased phosphorylation of the AKT pathway in HER2+ cells, which was postulated to be the primary mechanism of action of ibrutinib in this case. Moreover, it has been found that ibrutinib causes a reduction in the phosphorylation of EGFR, HER2 and HER3, thus inhibiting their downstream signaling. Lastly, the addition of the dactolisib, a PI3K/mTOR inhibitor, further reduced cell viability. This in vitro study was the first of its kind, proving the potential efficacy of ibrutinib in breast cancer, especially when combined with PI3K/mTOR inhibitors [86].

Sagiv-Barfi et al. [89] sought to investigate the efficacy of therapy composed of ibrutinib (Pharmacyclics LLC, Sunnyvale, CA, USA) and anti-PD-L1 antibodies (BioXcell, Lebanon, NH, USA) on several different cell lines, including the 4T1 cell line—a triple-negative breast cancer cell line. Importantly, no monotherapy has been previously identified as a feasible therapeutic option in 4T1-xenografted mice. In this case, consistently with previous observations, ibrutinib failed as a treatment option. The addition of the anti-PD-L1 antibody resulted in a significant reduction in tumor size, a reduction in the number of lung metastases and increased survival. These results suggest that the combination of PD1/PD-L1 blockade and ibrutinib might be an interesting option to be reviewed in drug-resistant triple-negative breast cancer [89].

Chen et al. [87] decided to focus on a different aspect of this issue. Here, the authors sought to address four main questions—how potent ibrutinib is in inhibiting HER2+ cells in vitro and in vivo, if ibrutinib inhibits these enzymes irreversibly, if it is possible to achieve an antitumor effect in vivo, and whether ibrutinib possesses any characteristic that could distinguish it from other EGFR family inhibitors. As discovered, ibrutinib (Pharmacyclics LLC, Sunnyvale, CA, USA) was capable of inhibiting the growth of several tumor cell lines (American Type Culture Collection, Manassas, VA, USA; Sigma-Aldrich, St. Louis, MO, USA; Asterland, Detroit, MI, USA) especially the HER2+ ones, with the authors evaluating its potency to be higher than those of gefitinib and lapatinib, although lower than that of afatinib and neratinib (ERBB family of inhibitors was obtained from Selleck Chemicals, Houston, TX, USA). The mechanism of this action was discovered to be similar to one of the HER2-inhibitors, with ibrutinib inhibiting the phosphorylation and downstream signaling of HER2 and EGFR in the affected cells. In xenograft studies, ibrutinib was discovered to cause a 90% reduction in tumor progression in MDA-MB-453 cells, which possess HER2+ mutation. It was calculated that such an effect might be achieved in humans with a dose of 560 mg/day, similar to the one used routinely in chronic lymphocytic leukemia therapy [87].

Stiff et al. [72] decided to focus on the influence of ibrutinib on MDSC—cells that, as mentioned previously, are linked to the loss of immune effector cell function and reduced efficacy of immune-based cancer therapies. The authors discovered that the treatment with ibrutinib resulted in decreased nitric oxide production and cell migration. Furthermore, ibrutinib in vivo inhibited the generation of human MDSC and reduced mRNA expression of indoleamine 2,3-dioxygenase, an immunosuppressive factor. In mice, xenografted with a triple-negative breast cancer cell line EMT6, ibrutinib significantly reduced the frequency of MDSC in the spleen and tumor. Furthermore, consistent with the Sagiv-Barfi et al. [89] study, the authors found therapy composed of ibrutinib and PD-L1-inhibitors to show a synergistic effect [72].

In 2013, Eifert et al. [46] reported a novel isoform of BTK, called BTK-C, to protect breast cancer cells from apoptosis. Later, in 2016, Wang et al. [42] continued this study and found that ibrutinib (ChemieTek, Indianapolis, IN, USA) decreases cancer cell (American Type Culture Collection, Manassas, VA, USA) survival and prevents drug resistance, with HER2+ cells being significantly more sensitive to this agent than luminal and triple-negative ones. Consistently with the Grabinski and Ewald study, Wang et al. [42] described ibrutinib to inhibit the activation of EGFR, HER2, HER3 and HER4, consequently blocking the activation of downstream pathways, with all these observations being later confirmed in the in vivo evaluation of the xenografted mice [42].

Using orthotopic mice, Varikuti et al. [90] sought to evaluate the progression and metastasis of breast cancer. The authors compared the aforementioned factors between a group of ibrutinib (Pharmacyclics LLC, Sunnyvale, CA, USA)-receiving mice and a placebo-receiving group. As discovered, mice treated with this BTKi exhibited a significant reduction in tumor progression and tumor weight. Furthermore, ibrutinib-treated mice had spleens and tumors containing significantly more mature dendritic cells (DC) and less MDSCs than the placebo group. Authors confirmed ex vivo that ibrutinib-treated MDSCs switched their phenotype to mature dendritic cells and had a significantly enhanced expression of major histocompatibility complex II (MHC II). Lastly, the treatment with ibrutinib significantly promoted the proliferation of the T-cells, leading to the induction of Th1 and the CD8+ T cell antitumor response [90].

Prabaharan et al. [88] evaluated the ibrutinib’s (Selleck Chemicals, Houston, TX, USA) effect on two HER2+ cell lines—BT474 and SKBR3 (American Type Culture Collection, Manassas, VA, USA). As discovered, this BTKi was capable of inducing changes in nuclear morphology and causing apoptosis via the caspase-dependent extrinsic apoptosis pathway. Furthermore, treatment with ibrutinib resulted in the upregulation of STAT3 and downregulation of p21. Thus, the authors proposed the upregulation of STAT3 to be a passive response, resulting from the induction of DNA damage and downregulation of phosphorylated p21, promoting cell cycle arrest and apoptosis in breast cancer cell lines. They proposed STAT3 inhibitors as a potentially excellent option for combination therapy with ibrutinib [88].

Hong et al. [82] focused on, among others, stage III/IV HER2+ and triple-negative breast cancer treated with an ibrutinib-durvalumab regime. Nevertheless, with 3% (n = 1) of the patients responding to such therapy, a mOS of 4.2 months (95% CI: 3.4–7.4 months) and a mPFS of 1.7 months (95% CI: 1.5–1.8 months), the results could be only described as poor. Similar to the observations regarding NSCLC patients, all (n = 45) breast cancer patients suffered AE as a result of such therapy, with the vast majority of them (n = 35; 78%) experiencing AE of grade 3 or above [82].

As observed in the studies described above, ibrutinib induces a diversity of anti-tumor effects in breast cancer. Therefore, we believe that of the studies described in this article, the rationale behind the feasibility of ibrutinib in breast cancer treatment is among the strongest ones. Nevertheless, the observations made in the present study regarding lung cancer are still applicable. To use ibrutinib as a therapeutic option in this malignancy, the discrepancy between in vitro and in vivo pharmacokinetics of ibrutinib needs to be overcome. Consequently, as of now, such therapy cannot be recommended.

### 3.5. Pancreatic Cancer

Massó-Vallés et al. [76] sought to evaluate the efficacy of ibrutinib in pancreatic ductal adenocarcinoma (PDAC). The authors discovered a reduction of 67.8 ± 29.5% in the cell mitotic rate, as measured by Ki67 expression, in the *p53^ER/ER^; LSLKRas^G12D^; Pdx1-cre* mice model. Furthermore, combined therapy composed of ibrutinib with gemcitabine—a drug routinely used to treat pancreatic cancer—resulted in decreased toxicity compared to standard gemcitabine monotherapy [76].

Tan et al. [91] focused on ibrutinib’s (Selleck Chemicals, Houston, TX, USA) ability to radiosensitize rather than on the feasibility of using it as a treatment option on its own. Here, the BTKi was found to enhance the effect of radiotherapy with a sensitization enhancement ratio (SER) of 1.34 and 1.68 in BXPC3 and Capan2 cells (Stem Cell Bank, Chinese Academy of Sciences), respectively. Furthermore, as measured through flow cytometry, ibrutinib combined with radiotherapy-induced G2/M arrest and cell apoptosis. To investigate the mechanism of this action, the authors evaluated EGFR and AKT/mTOR signaling pathways using western blotting. As discovered, ibrutinib decreased the phosphorylation of EGFR and reversed the upregulation of p-AKT and downstream genes induced by radiation. Lastly, consistent with the previous study, ibrutinib was found to inhibit the proliferation of cancer cells. As such, ibrutinib was discovered to be a potentially excellent radiosensitizer in pancreatic cancer, with the proliferation-inhibiting characteristic being a much welcome addition [91].

Similar to a study performed by Zhang et al. [109] regarding lung cancer, Tan et al. [111] decided to evaluate the efficacy of the previously mentioned Ibr-7. The authors treated PANC-1 and Capan2 (Stem Cell Bank, Chinese Academy of Sciences, Shanghai, China) pancreatic cancer cell lines in vitro with Ibr-7 (Hangzhou Hezheng Pharmaceutical Co., Ltd., Hangzhou, China) and ibrutinib (Selleck Chemicals, Houston, TX, USA). In the CCK-8 assay, this agent was found to inhibit cancer cell growth much more effectively than ibrutinib—the IC_50_ value of Ibr-7 was approximately one-tenth of the IC_50_ of ibrutinib. In the clonogenic survival assay, the authors evaluated the radiosensitivity of Ibr-7-treated cancer cells similarly to Tan et al.’s [81] study. Ibr-7 was found to induce radiosensitivity in both PANC-1 and Capan2 cell lines, with SERs of 1.63 and 1.59, respectively. Furthermore, Ibr-7 was found to induce G2/M arrest, increase radiation-induced apoptosis and elicit the damage done to the cancer cell DNA by such therapy. Consistently with the previous study, Ibr-7 might be an excellent radiosensitizer in pancreatic cancer [111]. Nevertheless, as mentioned previously, this agent has not been approved for use on humans, meaning that the current discussion regarding its feasibility as a treatment option is purely theoretical.

Nevertheless, consistently with our previous remarks regarding lung and breast cancer, Hong et al. [82] disproved the feasibility of ibrutinib in stage-III/IV pancreatic ductal adenocarcinoma (PDAC). In the third part of this study, scientists evaluated the efficacy of ibrutinib-durvalumab therapy in patients diagnosed with pancreatic adenocarcinoma. With 2% (n = 1) of the patients responding to such treatment, a mOS of 4.2 months (95% CI: 2.6–6.4 months) and a mPFS of 1.7 months (95% CI: 1.6–1.8 months), as well as all (n = 45) breast cancer patients suffering AE (grade 3 or above: n = 38; 78%), such therapy was once again deemed not only ineffective but also conducive to unnecessary side effects [82].

The most important study regarding the efficacy of ibrutinib in pancreatic cancer is arguably the one conducted by Tempero et al. [92] in phase III of the RESOLVE study. The authors sought to evaluate ibrutinib (IMBRUVICA^®^) in combination with nab-paclitaxel and gemcitabine (nab-P/GCB) as a first-line treatment in patients with metastatic PDAC. Patients were divided into two groups—both receiving nab-paclitaxel and gemcitabine, with the first receiving ibrutinib (n = 211) additionally, while the other received a placebo (n = 213). Here, the authors reported that ibrutinib did not affect the primary endpoint of overall survival (95% CI: 0.903–1.363), while decreasing the progression-free survival—mPFS in (95% CI: 1.277-1.916; ibrutinib + nab-P/GCB—5.32 months; placebo + nab-P/GCB—6.01 months). Considering these numbers, although the occurrence of grade-3 AEs was similar in the ibrutinib + nab-P/GCB and placebo + nab-P/GCB (86% vs. 87%, respectively), due to the decrease in the mPFS in the ibrutinib + nab-P/GCB group, we strongly discourage such therapy [92].

We hereby suspect that the difference in observations between preclinical and clinical studies most likely comes from the aforementioned discrepancy in the in vitro and in vivo pharmacokinetics. In the future, ibrutinib might once again re-emerge as a potentially feasible option in this type of malignancy if the discussed issue is overcome. For now, we believe that we should refrain from further clinical trials regarding the use of ibrutinib in pancreatic cancer.

### 3.6. Gastric Cancer

To the best of the authors’ knowledge, the only study regarding the efficacy of ibrutinib in gastric cancer is the one conducted by Wang et al. [93]. Their study consisted of two mains parts—in vitro evaluation of MGC-803, BGC-823, SGC7901, MKN-45 and MKN-28 gastric cancer cell lines (American Type Culture Collection, Manassas, VA, USA) treated with ibrutinib, and the assessment of the inhibition of the MKN-45 and BGC-823 xenograft tumor growth by ibrutinib. The authors discovered that BTK caused a reduction in the tumor volume, with no significant toxicities being observed. Lastly, ibrutinib was observed to act as a chemosensitizer for docetaxel, showing the synergistic effect of such therapy [93]. 

Although we believe these results do not justify using ibrutinib as an anti-cancer option in gastric cancer in clinical trials, using this BTKi as a chemosensitizer seems significantly more viable. We propose further investigations to focus on this trait of ibrutinib. 

### 3.7. Colon Cancer

Sagiv-Barfi et al.’s [89] study evaluated the effects of ibrutinib on the CT26 colon cancer cell line. Consistent with our previous remarks regarding breast cancer, ibrutinib combined with a non-specified PD-L1-inhibitor was an effective option in CT26-xenografted mice, capable of curing colon cancer entirely. Interestingly, the mice subsequently displayed a long-term memory, rejecting CT26 tumors upon reimplantation [89].

Grassilli et al. [94] discovered a novel isoform of BTK, named p65BTK, which is abundantly expressed in several colorectal cancer (CRC) lines (ATCC, LGC Standards, Sesto San Giovanni, Italy; Johns Hopkins University, Baltimore, MD, USA; Deutsche Sammlung von Mikroorganismen und Zellkulturen GmbH Braunschweig, Germany) and tumor tissue samples. The authors found p65BTK to be strongly involved with the RAS/ERK pathway, with the overexpression of p65BTK in colon cancer correlating with ERK1/2 activation. In the in vitro evaluation, the inhibition of this isoform decreased the growth and survival of cancer cells, thus serving as a rationale behind the potential feasibility of BTK inhibitors in this type of cancer [94].

Lavitrano et al. [49] decided to further investigate the role of p65BTK. Using drug-resistant *TP53*-null CRC cells (Johns Hopkins University, Baltimore, MD, USA; ATCC, LGC Standards, Sesto San Giovanni, Italy) in vitro, the authors discovered that the silencing or inhibition of p65BTK overcame the resistance of cancer to 5-fluorouracil, which was further confirmed in the ex vivo and in vivo evaluations. What is more, a significant reduction in xenografted tumor growth was also observed [49].

Kim et al. [95] sought to evaluate the efficacy of using ibrutinib (IMBRUVICA^®^) combined with pembrolizumab as a treatment option. Unfortunately, although the authors observed a significantly better safety profile than the Hong et al. [82] and the Tempero et al. [92] studies, with 16 (42%) patients experiencing a grade 3/4 AE, ibrutinib failed as a therapeutic option. The authors observed a mPFS of 1.4 months (95% CI: 1.4–1.5) and a mOS of 6.6 months (95% CI: 4.3–12.2). The authors of this study decided that, due to the limited efficacy of ibrutinib as a treatment option in CRC cancer, such therapy does not warrant further research of this combination [95].

However, ibrutinib still poses an interesting therapeutic option for colon cancer. We believe that the ability to reject the subsequent tumor implantations, as observed by Sagiv-Barfi et al. [89], is a particularly interesting trait to be investigated in the future. Furthermore, as demonstrated by Grassilli et al. [94] and Lavitrano et al. [49], targeting p65BTK could serve as a novel therapeutic approach in colon cancer patients, capable of inducing a response to the 5-fluorouracil of drug-resistant colon cancer. However, due to the lack of effects of treatment with this drug, as described by Kim et al. [95], we suggest further research to focus on ibrutinib as a chemosensitizer and not as a therapeutic option in itself.

### 3.8. Prostate Cancer

To the best of the authors’ knowledge, the only study evaluating the efficacy of ibrutinib in prostate cancer is the one conducted by Zhu et al. [96]. In this study, the authors discovered a strong expression of BTK detected in the prostate cancer tissues, especially in the tissue samples of tumors sampled from prostate cancer patients with bone metastases. As such, ibrutinib (Cell Signaling Technology, Danvers, MA, USA) was evaluated as a potentially feasible agent for prostate cancer. Indeed, this BTKi was discovered to significantly inhibit cell proliferation, migration, and invasion of prostate cancer cells. Furthermore, ibrutinib decreased the synthesis of matrix metalloproteinases-2 and -9 (MMP-2 and MMP-9)—endopeptidases, of which overexpression is associated with increased invasiveness and severity of prostate cancer [96,112,113]. 

We believe that ibrutinib might be an interesting option to be further reviewed in in vivo preclinical studies regarding prostate cancer. As of now, the evidence is too limited to make any firm statements.

### 3.9. Neuroendocrine Tumors

Al-Toubah et al. [97] sought to evaluate the efficacy of the treatment with ibrutinib (IMBRUVICA^®^) in a clinical trial involving 15 patients diagnosed with gastrointestinal/lung neuroendocrine tumors and 5 diagnosed with pancreatic neuroendocrine tumors. Unfortunately, such treatment was declared ineffective, with 0% of patients responding to such therapy and a mPFS of 3 months (95% CI, 2.8–5.8 months) [97].

Due to the lack of results described by Al-Toubah et al. [97], we believe that although more studies are required, as of now, the focus should be shifted to the preclinical evaluation of ibrutinib in neuroendocrine tumors.

### 3.10. Glioblastoma

Wei et al. [98] investigated in vitro the effects of ibrutinib on U87MG and DBTRG-05MG cell lines (American Type Culture Collection) as well as xenograft animal studies performed with U87MG parental and BTK-silenced U87MG cells. In their study, the authors observed that ibrutinib (Selleck Chemicals, Taiwan) in vitro suppressed the tumorigenesis of glioblastoma cells. Significantly, a combination of ibrutinib with temozolomide (Selleck Chemicals, Taiwan), an alkylating agent, suppressed the GBM sphere-forming ability and, consequently, the stemness and metastatic potential more potently than either of these two drugs alone. Furthermore, these results were further confirmed in the xenograft animal study. The authors found that both ibrutinib/temozolomide and ibrutinib alone therapies exerted a more potent anti-tumorigenic effect than temozolomide alone [98].

Wang et al. [99] sought to investigate a similar subject, albeit focusing on the evaluation of LN229, U87, T98, and U251 cell lines (American Type Culture Collection, Shanghai, China) in vitro and U87 in a xenograft animal study. As discovered, ibrutinib (Selleck Chemicals, Houston, TX, USA) decreased the cellular proliferation and migration of glioblastoma cells, as well as increased the apoptosis and autophagy of the LN229 and U87 cell lines. While the overexpression of Akt protein has been discovered to decrease this process, the inhibition of Akt protein by LY294002, a PI3K inhibitor, resulted in an increased apoptosis and autophagy of the aforementioned LN229 and U87 cell lines. Lastly, the anti-cancer capabilities of ibrutinib in vitro and in vivo have been further increased by the inhibition of autophagy by 3-methyladenine or *Atg7* targeting with small interfering RNA [99].

We believe that glioblastoma poses an interesting target of the therapy with ibrutinib, as both studies are promising and encourage further research. Similar to the majority of the previous types of malignancies, ibrutinib seems to be moderately effective on its own, and the therapy composed of this BTKi and other agents, such as PI3K inhibitor LY294002 and temozolomide, shows considerably more promise. We believe that initial research proved ibrutinib to be a potentially feasible option as a part of a multidrug regime. Nevertheless, it is crucial to mention that as both of these studies were preclinical, further research is required to accurately evaluate the efficacy of ibrutinib in glioblastoma. 

## 4. Side Effects of the Therapy with Ibrutinib

Due to the broad and multidirectional activity profile of ibrutinib, several side effects can be expected to result from the activity of these agents. Although they usually occur in a mild or moderate intensity (I–III degree), one should be aware of the possibility of their occurrence with life-threatening severity. 

The most common undesirable effects associated with ibrutinib therapy are hair and nail disorders, occurring in 26% and 66% of patients, respectively. In hair disorders, reactions are characterized by a straightening and thinning of the hair, while nail disorders most often include brittle or splitting fingernails [114]. Other dermatological manifestations, which occur in 2–27% of patients during ibrutinib treatment, are most often related to the formation of petechiae, bruising or rash [115,116,117]. Due to its morphology, the latter may often resemble leukocytoclastic vasculitis [118]. Casuistically, the therapy with ibrutinib may result in the development of a severe allergy coexisting with grade-III rash [118]. 

The treatment with ibrutinib is characterized by an increased risk of bleeding, demonstrated in randomized clinical trials [115,119]. The overall bleeding rate was calculated as 20.8 per 100 patient-years (95% CI 19.1–22.1), and an overall relative risk of any bleeding was found to be 2.72 (95% CI 1.62–6.58) [120]. Typically, in clinical trials, the risk of significant bleeding remained low, with a predominance of grade-I–II bleeding in the form of petechiae or contusions [121,122]. It was reported that the rate of severe episodes, including subdural hematomas, gastrointestinal bleeding or hematuria, increased in the early phases of clinical trials [115,119]. Despite the association of the role of BTKi with the platelet signaling processes by Glycoprotein 1b (Gp1B) and Glycoprotein IV (GpVI), molecules mediating platelet aggregation and adhesion via the von Willebrand factor, the influence of BTKi on the risk of bleeding is still unclear [123]. It is indicated that there is no risk of increased bleeding in patients with X-linked agammaglobulinemia, despite the lack of functional BTK. As such, bleeding resulting from the use of ibrutinib cannot be explained by just the inhibition of this kinase. It has been suggested that in the course of chronic lymphocytic leukemia, platelet dysfunction occurs due to impairment of ADP and collagen-dependent adhesion. Lipsky et al. noticed a reduced risk of bleeding 6 months after the start of therapy, which was explained by an improving status of the patient resulting from the use of ibrutinib [124].

Another side effect that causes up to 32% of discontinuation of therapy is atrial fibrillation (AF), which can cause strokes, cardiomyopathy and mortality [125,126,127]. The mechanism by which ibrutinib causes AF has not been established, but attention is drawn to the connections between BTK, TEC and the PI3K-Akt pathway [128]. Although the use of tyrosine kinase inhibitors is sometimes the cause of QTc prolongation, no such correlation is observed in the case of ibrutinib use [129]. One meta-analysis found an increased incidence of AF in the group of patients treated with ibrutinib compared to the population treated with other treatments, and the incidence rate was 3.3/100 person-years (95% CI 2.5–4.1) vs. 0.8/100 person-years (95% CI 0.32–1.6), respectively [130]. Worth mentioning are the risk groups for this side effect: older age (≥65), male gender, hypertension, diabetes, heart disease and left atrial enlargement [131,132].

Among other cardiological side effects, ventricular arrhythmias (ventricular tachycardia and ventricular fibrillation) and sudden cardiac death are casuistic. The use of ibrutinib may also lead to hypertension in up to 25% of patients, leading to the occurrence of AF [133,134,135].

Worth mentioning are less frequent but significant complications, such as an increased risk of opportunistic infections (i.e., *Pneumocystis jiroveci*), including fungal infections (i.e., *Aspergillus* spp.), primarily associated with neutropenia, the use of corticosteroids and other forms of anti-cancer therapy, such as immunotherapy [122,136,137]. Lastly, hepatitis B virus (HBV) reactivation, described by Hammond et al., is another serious casuistically reported infectious complication [138]. 

Due to its profile of action, the use of ibrutinib may also induce the occurrence of cytopenia, in particular anemia, thrombocytopenia and neutropenia (a frequency of 5%, 5%, and 10–17%, respectively), typically occurring in the first months of treatment [116,121,122,139].

## 5. Conclusions

Since its first approval in 2007, ibrutinib has established itself as an efficient therapeutic option in several hematological malignancies, but its feasibility might not end there. Numerous clinical trials are currently underway (Table 3), with published data at present being of particular interest. Several studies have evaluated its use in anti-solid tumor therapy, discovering significant discrepancies between the preclinical and clinical studies. As suspected, these differences might be related to the use of this drug in the non-commercial formulation, with its in vivo pharmacokinetics being designed to affect the cells of the B-cell lineage. We believe that the development of alternative drug formulations and the addition of appropriate adjuvants might result in a significantly increased effectiveness of anti-solid tumor therapy with ibrutinib. If those issues were to be overcome, ibrutinib might emerge as an effective therapeutic option. Still, due to its proven synergistic effect with several established monoclonal antibodies, we believe that future studies should instead focus on the feasibility of using this BTKi as a part of a multi-drug regime rather than as a medication on its own. While the majority of the preclinical studies proved ibrutinib to be an effective treatment option for solid tumors, all of the clinical trials contradicted this observation.

## Figures and Tables

**Table 1:** Kinases prone to the inhibition by ibrutinib.

Kinase	IC_50_ (nM)
BTK	0.5
BLK	0.5
BMX	0.8
CSK	2.3
BRK	3.3
HCK	3.7
EGFR	5.6
YES	6.5
HER2	9.4
ITK	10.7
JAK3	16.1
FRK	29.2
LCK	33.2
RET	36.5
FLT3	73
TEC	78
RIPK2	152
c-SRC	171
LYN	200
PDGFRα	718
mTOR	4253

Adapted from Honigberg et al., 2010 [11] and Molina-Cerrillo et al., 2017 [48]. BTK, Bruton’s tyrosine kinase; BLK, B-lymphocyte kinase; BMX, bone marrow tyrosine kinase on chromosome X; CSK, C-terminal Src kinase; BRK, breast tumor kinase; HCK, hematopoietic cell kinase; EGFR, epidermal growth factor receptor 1; YES, proto-oncogene tyrosine-protein kinase Yes; HER2; epidermal growth factor receptor 2; ITK, IL-2 inducible T-cell kinase; JAK3, Janus kinase 3; FRK, Fyn-related kinase; LCK, Lymphocyte-specific protein tyrosine kinase; RET, rearranged during transfection kinase; FLT3, fms like tyrosine kinase 3; TEC, tyrosine kinase expressed in hepatocellular carcinoma; RIPK2, receptor interacting serine/threonine kinase 2; c-SRC, proto-oncogene tyrosine-protein kinase Src; LYN, Lck/Yes novel tyrosine kinase; PGDFRα, platelet-derived growth factor receptor α; mTOR; mechanistic target of rapamycin; IC_50_, half-maximal inhibitory concentration.

**Table 2:** Ibrutinib in studies: tumor specific evaluation.

Malignancy	Ibrutinib in Preclinical Studies	Ibrutinib in Clinical Trials
Lung cancer	Increased ST in animal studies [79] Effective towards mutant EGFR cell lines; synergistic effect with MEK-inhibitor in vitro [80] Suppressed tumor cell proliferation, migration and invasion [81]	No effect towards NSCLC [82]
Endometrial cancer	Suppressed growth of the tumor; higher activity towards endometrioid adenocarcinoma with squamous differentiation than towards clear cell adenocarcinoma [83]	n.a.
Ovarian cancer	Platinum sensitizer [84] No activity towards endometrial clear cell adenocarcinoma [85]	n.a.
Breast cancer	Activity towards HER2+ cell lines [46,86,87,88] Synergistic effect with PI3K/mTOR inhibitor [86] Synergistic effect with PD1/PD-L1 inhibitor in triple negative cell line [89] Inhibited generation of MDSC [72,90] Reduced mRNA expression of indoleamine 2,3-dioxygenase [90] Reduced tumor mass and progression [87]	Very poor (OR–3%) activity; mPFS—4.2 months; mOS—1.7 months [82]
Pancreatic cancer	Reduced proliferation [76] Radiosensitizer [91] Ibrutinib reduced toxicity caused by gemcitabine [76]	Decrease in mPFS as compared to placebo [92]
Gastric cancer	Suppressed growth and survival of cancer cells; chemosensitizer for docetaxel [93]	n.a.
Colon cancer	Suppressed growth and survival of cancer cells [94] Synergistic effect with PD-L1 inhibitor [89] Chemosensitizer for 5-fluorouracil [49]	Well tolerated, but with limited anti-cancer activity; mPFS—1.4 months; mOS—6.6 months [95]
Prostate cancer	Suppressed tumor cell proliferation, migration, and invasion [96]	n.a.
Neuroendocrine tumors	n.a.	No activity [97]
Glioblastoma	Suppressed tumor cell proliferation, migration and invasion [98,99] Synergistic effect with PI3K inhibitor [99]	n.a.

MDSC, myeloid-derived suppressor cells; mOS, median overall survival; mPFS, median progression free survival; n.a., not applicable; NSCLC, non-small cell lung cancer; OR, overall response; ST, survival time.

**Table 3:** Ibrutinib in solid tumors: currently undergoing clinical trials.

Neoplasm	Comedication	Phase	NCT
HER2+ breast cancer	Trastuzumab	I/II	NCT03379428
Prostate cancer	n.a.	II	NCT02643667
Colon cancer	Pembrolizumab	I/II	NCT03332498
Melanoma	n.a.	II	NCT02581930
Ependymoma Medulloblastoma Glioblastoma PNET	Indoximod Cyclophosphamide Etoposide	I	NCT05106296
Solid tumors	Nivolumab	I	NCT03525925
Head and neck squamous cell carcinoma	Nivolumab Cetuximab	II	NCT03646461

PNET, Primitive Neuroectodermal Tumor; n.a., not applicable.
